# Lightning Strike

**DOI:** 10.21980/J8SD2M

**Published:** 2022-01-15

**Authors:** Thomas Powell, Aubri Charnigo, Jennifer Yee

**Affiliations:** * The Ohio State University, Department of Emergency Medicine, Columbus, OH

## Abstract

**Audience:**

This scenario was developed to educate emergency medicine residents on the various presentations and management of a patient struck by lightning.

**Introduction:**

Annually, there are approximately 1.4 billon lightning strikes around the world; of these, an estimated 24,000 strikes cause significant injury or death.^1^ In the United States, there are approximately 400 lightning-related injuries every year resulting in 40 average annual deaths.^1^ Although only one in approximately 14,000 people will ever be struck by lightning, this still represents a significant injury mechanism for which emergency department providers must be prepared.^2^ Lightning is formed by static electricity built up due to ice crystals in clouds which creates a differential charge between the cloud and another object, such as the ground. Approximately one in every five lightning strikes is a cloud-to-ground strike which can result in injury or death. Lightning current flows may be as high as 100,000 amperes; this is survived 90% of the time only because the strong current of the bolt is applied in a very small timeframe, limiting the amount of energy transferred.^3^ Even so, with such large amperages, substantial injuries or death are possible. Not limited to a single mechanism, lightning can harm people in a variety of ways, including a direct strike, side-splash, ground current or upward streamers from the ground, or cause blast-type injury.^2^ The large electric currents involved can generate non-perfusing cardiac rhythms resulting in death if the patient is not immediately resuscitated through cardiopulmonary resuscitation (CPR) techniques with respiratory support.^2^

**Educational Objectives:**

At the conclusion of the simulation session, learners will be able to: 1) Describe how to evaluate for scene safety in an outdoor space during a thunderstorm, 2) Obtain a relevant focused physical examination of the lightning strike patient, 3) Describe the various manifestations of thermo-electric injury, 4) Discuss the management of the lightning strike patient, including treatment and disposition, 5) Outline the principles of reverse triage for lightning strike patients, and 6) Describe long-term complications of lightning strike injuries.

**Educational Methods:**

This session was conducted using a simulation scenario with a mix of high-fidelity manikins and standardized patients followed by a debriefing session on the presentation, differential diagnosis, and management of lightning strike patients. Debriefing methods may be left to the discretion of participants, but the authors have utilized advocacy-inquiry techniques. This scenario may also be run as an oral board examination case.

**Research Methods:**

The residents are provided a survey at the completion of the debriefing session to rate different aspects of the simulation, as well as to provide qualitative feedback on the scenario. This survey is specific to the local institution’s simulation center.

**Results:**

Feedback from the residents was overwhelmingly positive, although several learners struggled with identifying Lichtenberg figures and keraunoparalysis either due to the low-light setting, unfamiliarity of the pathology, or that the depictions were not as expected. The subsequent debriefings allowed for multiple areas of discussion. Debriefing topics included the comparing and contrasting low voltage/high voltage/lightning strike injuries, possible clinical presentations of the lightning strike patient, reverse triage principles, categorizing blast injuries, discussion of disposition, and the determination of prehospital scene safety.

The local institution’s simulation center feedback form is based on the Center of Medical Simulation’s Debriefing Assessment for Simulation in Healthcare (DASH) Student Version Short Form^4^ with the inclusion of required qualitative feedback if an element was scored less than a 6 or 7. Thirty-one learners completed a feedback form. This session received all 6 and 7 scores (consistently effective/very good and extremely effective/outstanding, respectively) other than one isolated 5 score. The statement, “Before the simulation, the instructor set the stage for an engaging learning experience,” received the lowest average score with 6.81, while “The instructor structured the debriefing in an organized way” received an average score of 6.94.

The form also includes an area for general feedback about the case at the end. Illustrative examples of feedback include: “Absolutely loved this sim. Tested multiple aspects of massCal care. Communication, critical care, scene safety, etc., nailed it,” and “Very engaging and fun with a lot (of) good debriefing.”

**Discussion:**

This is an easily reproducible method for reviewing management of the lightning strike patient. Faculty may choose to use a combination of high- or low-fidelity manikins, task trainers, standardized patients, or confederate actors/volunteers as patients. There are multiple potential presentations and complications of the lightning strike patient to further customize the experience for learners’ needs. For those who are looking to scale down the scenario, victims may be limited to one or two individuals, using whatever preferred mixture of manikins or standardized patients is needed or desired.

**Topics:**

Medical simulation, lightning strike patient, thermo-electrical burn, wilderness first-aid, blast injuries, wilderness medicine, emergency medicine, austere medicine.

## USER GUIDE

**Table:** 

List of Resources:	
Abstract	78
User Guide	80
Instructor Materials	82
Instructor Materials—Patient Callaway	87
Operator Materials—Patient Callaway	91
Instructor Materials—Patient Mizuno	92
Operator Materials— Patient Mizuno	94
Instructor Materials—Patient Ping	94
Operator Materials— Patient Ping	97
Debriefing and Evaluation Pearls	98
Simulation Assessment	102

**Learner Audience:**

Medical students, interns, junior residents, senior residents, emergency medical service (ems)/first responders

**Time Required for Implementation:**

Instructor Preparation: 30 minutes

Time for case: 20 minutes

Time for debriefing: 40 minutes

**Recommended Number of Learners per Instructor:**

3–4

**Topics:**

Medical simulation, lightning strike patient, thermoelectrical burn, wilderness first-aid, blast injuries, wilderness medicine, emergency medicine, austere medicine.

**Objectives:**

By the end of this simulation session, the learner will be able to:

1. Describe how to evaluate for scene safety in an outdoor space during a thunderstorm
2. Obtain a relevant focused physical examination of the lightning strike patient
3. Describe the various manifestations of thermoelectric injury
4. Discuss the management of the lightning strike patient, including treatment and disposition.
5. Outline the principles of reverse triage for lightning strike patients
6. Describe long-term complications of lightning strike injuries

### Linked objectives and methods

Patients who have suffered a lightning strike require prompt evaluation and may require immediate prioritization of care for CPR. In this case, providers will review the following high-yield aspects of lightning injuries. Providers will learn how to assess scene safety (Objective 1), how to quickly assess and diagnose the lightning strike patient with an appropriately focused physical exam (Objective 2) and evaluate for common injury patterns (Objective 3). Participants will perform reverse triage, administer appropriate initial resuscitative measures (Objective 4 and 5) and provide post-strike resuscitation, disposition, and instructions for follow-up care (Objective 4 and 6). This simulation scenario allows learners to reinforce their lightning strike patient management skills in a physically and psychologically safe learning environment and then receive formative feedback on their performance.

### Results and tips for successful implementation

This simulation was written to be performed as a high-fidelity simulation scenario but also may be used as a mock oral board case.

The case was written for emergency medicine residents. This lightning strike simulation case was conducted for approximately 35 emergency medicine residents during October-December 2021. The residents found this case challenging since lightning strike injuries are low-frequency cases in the emergency department and the residents had minimal prior clinical exposure to this injury. Multiple residents interpreted the patient with a cold blue mottled leg as compartment syndrome, despite verbalizing that the patient’s leg compartments were soft and compressible; having the patient emphasize that they had a normal appearing functional leg just a few minutes ago may help steer learners towards the correct keraunoparalysis diagnosis. Prior to running the simulation scenario, learners were surveyed to ensure that they did not have a medical diagnosis that they wished to disclose that would affect their ability to be around strobing lights. For those who are looking to scale down the scenario, victims may be limited to one or two individuals, using whatever preferred mixture of manikins or standardized patients is needed or desired.

The local institution’s simulation center feedback form is based on the Center of Medical Simulation’s Debriefing Assessment for Simulation in Healthcare (DASH) Student Version Short Form^4^ with the inclusion of required qualitative feedback if an element was scored less than a 6 or 7. Thirty-one learners completed a feedback form. This session received all 6 and 7 scores (consistently effective/very good and extremely effective/outstanding, respectively) other than one isolated 5 score. The statement, “Before the simulation, the instructor set the stage for an engaging learning experience,” received the lowest average score with 6.81, while “The instructor structured the debriefing in an organized way” received an average score of 6.94.

## Supplementary Information



## Figures and Tables

**Table:** 

#1	Lichtenberg Figure on Skin

**Table:** 

**Vitals:**	Heart Rate (HR) 0	Blood Pressure (BP) unmeasurable	Respiratory Rate (RR) 0
	Temperature (T) normothermic to touch	Oxygen Saturation (O_2_Sat) unmeasurable	

**Figure:**
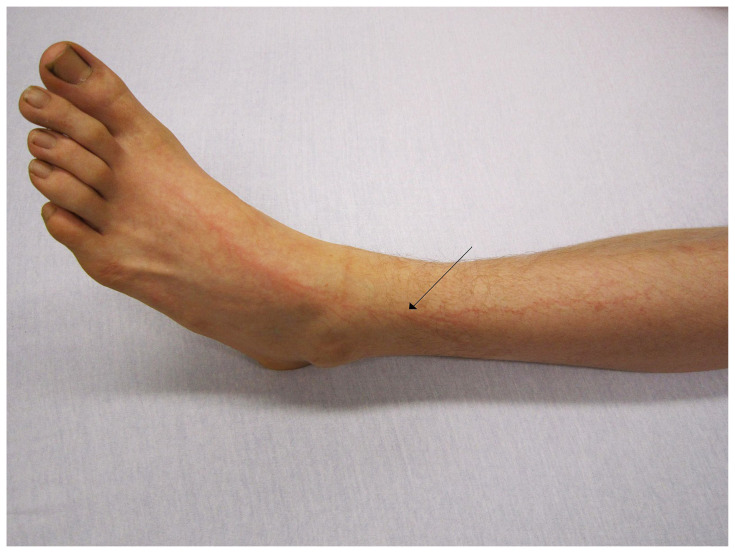


**Table:** SIMULATION EVENTS TABLE:

Minute (state)	Participant action/trigger	Patient status (simulator response) & operator prompts
0:00 (Baseline)	Patient Callaway struck by lightning	Learners should begin by assessing for scene safety, reviewing obtainable field initial vitals and assessing the patient
2:00	CPR should be initiated. An AED should be utilized to assist in the care of the patient with the pads properly placed	Patient unresponsive and in PEA once AED applied - CPR & ventilation initiated, AED demonstrates PEA
4:00	2^nd^ Pulse check	If the patient received CPR and rescue breaths: 2^nd^ pulse check confirms ROSC. AED now demonstrates sinus rhythm. Successful return to spontaneous circulation and breathing If the patient did not receive CPR and/or ventilation: 2^nd^ pulse check has no pulse, PEA on AED
6:00	Call for EMS transport	If second pulse check not performed by this point, ROSC will not be achieved. If rescue breaths not performed by this point, ROSC will not be achieved. If patient has now received CPR AND ventilation, ROSC will be achieved and the patient will begin to wake up and talk to rescuers

**Table:** 

**Vitals:**	Heart Rate (HR) 120	Respiratory Rate (RR) 22
	Temperature (T) normothermic to touch other than right lower extremity	

**Table:** SIMULATION EVENTS TABLE:

Minute (state)	Participant action/trigger	Patient status (simulator response) & operator prompts
0:00 (Baseline)	Patient Mizuno struck by lightning	Learners should begin by assessing for scene safety, reviewing obtainable field initial vitals and assessing the patient, starting with patient Callaway or simultaneously with patient Callaway (if multiple learners)
2:00	Brief assessment should be completed. Exam findings identified and verbalized to team	- Patient found to have a pulseless cyanotic right lower extremity - Pt emotionally upset
4:00	Reassurance should be given	If an assessment is performed and reassurance given, patient starts to become more reasonable.
6:00	If reassurance not given, patient becomes hysterical	If reassurance given by four minutes, patient able to assist team by reassuring patient Ping. If Mizuno is not reassured, patient becomes hysterical

**Table:** 

**Vitals:**	Heart Rate (HR) 135	Respiratory Rate (RR) 20
	Temperature (T) normothermic to touch	

**Table:** SIMULATION EVENTS TABLE:

Minute (state)	Participant action/trigger	Patient status (simulator response) & operator prompts
0:00 (Baseline)	Patient Ping struck by lightning	Learners should begin by assessing for scene safety, reviewing obtainable field initial vitals and assessing the patient after prioritizing patient Callaway and Mizuno (or simultaneously with the other 2 patients if there are enough learners).
2:00	Brief assessment should be completed. Wound identified and verbalized to team; proximal tourniquet appropriately placed	Patient found to have a penetrating left arterial upper arm injury with active bleeding; tourniquet placed
4:00	Patient becomes less hysterical	If patient informed that his bleeding is controlled and does not have any other serious injuries, he will become more calm
6:00 -end	Pt becomes more hysterical if tourniquet not applied	If not treated, patient should become as distracting as possible. If assessed, treated appropriately, and told to remain calm, patient should do so

**Table:** 

**Critical Actions:** Discuss the case with patient’s husband Assess circulation, airway, and breathing on all three patients Treatment via reverse triage, prioritizing resuscitation of the unresponsive, pulseless patient Recognition of keraunoparalysis and educating the patient that this will self-resolve Application of a tourniquet to the penetrating arterial injury Calling Emergency Medical Services for disposition	0:00

**Table:** 

	Milestone	Did not achieve level 1	Level 1	Level 2	Level 3
1	**Emergency Stabilization (PC1)**	□ Did not achieve Level 1	□ Recognizes abnormal vital signs	□ Recognizes an unstable patient, requiring intervention Performs primary assessment Discerns data to formulate a diagnostic impression/plan	□ Manages and prioritizes critical actions in a critically ill patient Reassesses after implementing a stabilizing intervention
2	**Performance of focused history and physical (PC2)**	□ Did not achieve Level 1	□ Performs a reliable, comprehensive history and physical exam	□ Performs and communicates a focused history and physical exam based on chief complaint and urgent issues	□ Prioritizes essential components of history and physical exam given dynamic circumstances
3	**Diagnostic studies (PC3)**	□ Did not achieve Level 1	□ Determines the necessity of diagnostic studies	□ Orders appropriate diagnostic studies. Performs appropriate bedside diagnostic studies/procedures	□ Prioritizes essential testing Interprets results of diagnostic studies Reviews risks, benefits, contraindications, and alternatives to a diagnostic study or procedure
4	**Diagnosis (PC4)**	□ Did not achieve Level 1	□ Considers a list of potential diagnoses	□ Considers an appropriate list of potential diagnosis May or may not make correct diagnosis	□ Makes the appropriate diagnosis Considers other potential diagnoses, avoiding premature closure
5	**Pharmacotherapy (PC5)**	□ Did not achieve Level 1	□ Asks patient for drug allergies	□ Selects an medication for therapeutic intervention, consider potential adverse effects	□ Selects the most appropriate medication and understands mechanism of action, effect, and potential side effects Considers and recognizes drug-drug interactions
6	**Observation and reassessment (PC6)**	□ Did not achieve Level 1	□ Reevaluates patient at least one time during case	□ Reevaluates patient after most therapeutic interventions	□ Consistently evaluates the effectiveness of therapies at appropriate intervals
7	**Disposition (PC7)**	□ Did not achieve Level 1	□ Appropriately selects whether to admit or discharge the patient	□ Appropriately selects whether to admit or discharge Involves the expertise of some of the appropriate specialists	□ Educates the patient appropriately about their disposition Assigns patient to an appropriate level of care (ICU/Tele/Floor) Involves expertise of all appropriate specialists
9	**General Approach to Procedures (PC9)**	□ Did not achieve Level 1	□ Identifies pertinent anatomy and physiology for a procedure Uses appropriate Universal Precautions	□ Obtains informed consent Knows indications, contraindications, anatomic landmarks, equipment, anesthetic and procedural technique, and potential complications for common ED procedures	□ Determines a back-up strategy if initial attempts are unsuccessful Correctly interprets results of diagnostic procedure
20	**Professional Values (PROF1)**	□ Did not achieve Level 1	□ Demonstrates caring, honest behavior	□ Exhibits compassion, respect, sensitivity and responsiveness	□ Develops alternative care plans when patients’ personal beliefs and decisions preclude standard care
22	**Patient centered communication (ICS1)**	□ Did not achieve level 1	□ Establishes rapport and demonstrates empathy to patient (and family) Listens effectively	□ Elicits patient’s reason for seeking health care	□ Manages patient expectations in a manner that minimizes potential for stress, conflict, and misunderstanding. Effectively communicates with vulnerable populations, (at risk patients and families)
23	**Team management (ICS2)**	□ Did not achieve level 1	□ Recognizes other members of the patient care team during case (nurse, techs)	□ Communicates pertinent information to other healthcare colleagues	□ Communicates a clear, succinct, and appropriate handoff with specialists and other colleagues Communicates effectively with ancillary staff

## INSTRUCTOR MATERIALS

**Case Title:** Lightning Strike

**Case Description & Diagnosis (short synopsis):** Patients are Wilson Ping (30-year-old male; standardized patient/confederate), Taylor Callaway (35-year-old male; manikin), and Nike Mizuno (37-year-old male; standardized patient/confederate). Each patient has a non-contributory past medical history. They were playing a game of golf when they suffered a cloud-to-ground lightning strike in the middle of their gathering. Bystanders call the scenario participants to the scene to see if they can help. Fortunately, there is an automatic external defibrillator (AED) and a Stop-the-BleedR kit present at a nearby club house. Patient Ping has significant left arm bleeding with a penetrating wound. Patient Mizuno will have a paralyzed right lower extremity that is cyanotic and pulseless. Patient Callaway will be unresponsive and pulseless with fixed, dilated pupils and a Lichtenberg figure on his right upper extremity. Initial rhythm check with the automated external defibrillator (AED) on Callaway will demonstrate pulseless electrical activity (PEA). Ideal management is to perform reverse triage so that patient Callaway is immediately resuscitated using basic life support (BLS) including rescue ventilation and chest compressions. Patient Mizuno will be evaluated due to threat to limb before being determined to be experiencing keraunoparalysis, and patient Ping will have a tourniquet applied to his left upper extremity above a penetrating arterial injury. The treatment team should contact emergency medical services (EMS) to have the patients transported for further care. Non-ideal management is to try treat the keraunoparalysis patient first, delay care for the patient in cardiac arrest, or delay tourniquet placement. Finally, the providers should contact EMS crews for prompt disposition of the patients and be able to describe who should be prioritized for further care.

**Equipment or Props Needed:**

A high-fidelity manikin for patient Callaway

An AED device

A Stop the BleedR kit

Moulage to apply Lichtenberg figure to patient Callaway

Bleeding moulage with hand pump to depict a penetrating left upper extremity injury for patient Ping

Moulage to apply blue/gray skin with mottling to patient Mizuno to depict keraunoparalysis

**Additional equipment that may be added for fidelity**

Artificial grass

Golf clubs, tees, and golf balls to be placed around the scene

Lightning effects machine (authors used Morris Perfect Storm 2.0 Lightning and Thunder Effects Machine)

Monitor playing a looped video of a lightning storm

**Confederates needed:**

Two standardized patients or confederates

**Stimulus Inventory:**

**Background and brief information:** Patients Callaway, Mizuno, and Ping continued golfing throughout a thunderstorm. Bystanders called 9-1-1 when they saw them fall to the ground after a nearby lightning strike.

**Initial presentation Patient Callaway:**

Patient is Taylor Callaway, who is a 35-year-old male who presents as an unresponsive patient post lightning strike. He was playing a game of golf with two friends when a lightning strike from the approaching thunderstorm struck the group and rendered him unresponsive.

- Past medical history: unknown
- Past surgical history: unknown
- Medications: unknown
- Allergies: unknown
- Family history: unknown
- Vital signs:
  ○ Heart rate (HR) – pulseless. AED demonstrates pulseless electrical activity
  ○ Respiratory (Resp) rate – No spontaneous respirations
  ○ Temperature (Temp) – Normothermic to touch
- Weight: 220 pounds (lbs.), 100 kilograms (kg)

Assessment: Patient is dressed in golfing clothes. He is unresponsive, pulseless, and without spontaneous respirations. +Lichtenberg figure right upper extremity

**Initial presentation Patient Mizuno:**

Patient is Nike Mizuno, who is a 37-year-old male who presents with a cool, pulseless, cyanotic and paralyzed right lower extremity post lightning strike. He was playing a game of golf with two friends when a lightning strike from an approaching thunderstorm struck the group.

- Past medical history: Depression
- Past surgical history: none
- Medications: Sertraline 10 milligrams (mg) daily
- Allergies: none
- Family history: noncontributory
- Vital signs:
  ○ HR – 120, pulseless right lower extremity (pulselessness verbalized if checked by learners)
  ○ Resp rate – 22 breaths per minute
  ○ Normothermic to touch other than right lower extremity
- Weight: 200 lbs., 91 kg

Assessment: Patient is dressed in golfing clothes. He is disturbed by the paralysis in his right leg, which is blue and mottled in a circumferential pattern from the hip down.

**Initial presentation Patient Ping:**

Patient is Wilson Ping, who is a 30-year-old male who presents with a left arm penetrating arterial injury post lightning strike. He was playing a game of golf with two friends when a lightning strike from the approaching thunderstorm struck the group.

- Past medical history: None
- Past surgical history: eye surgery 3 years prior
- Medications: Daily multivitamin
- Allergies: none
- Family history: noncontributory
- Vital signs:
  ○ HR – 135, thready left radial pulse (verbalized if checked by learners)
  ○ Resp rate – 24 breaths per minute
  ○ Temp – Normothermic to touch
- Weight: 180 lbs., 82 kg

Assessment: Patient is dressed in golfing clothes. He has significant bleeding from a left upper arm penetrating injury, which ceases upon appropriate placement of a tourniquet (learners will place the tourniquet in the appropriate location, but it will not be tightened – facilitators will specifically observe placement).

**How the scene unfolds:** The learners are on a golf course where bystanders call them over after a lightning strike hit a group of three men. Learners rush over to assess the men and find the following scene: patient Ping has significant left arm bleeding with a penetrating arterial wound, patient Mizuno will have a paralyzed right lower extremity that is cyanotic and pulseless, and patient Callaway will be unresponsive and pulseless with fixed, dilated pupils and a Lichtenberg figure on his right upper extremity. The providers may be prompted that there is a nearby clubhouse with an AED and Stop the BleedR kit present. The providers must first check for scene safety. Once the scene is determined to be safe, they will assess the circulation, airway, and breathing of the patients in a manner consistent with reverse triage. Neither of the two responsive patients will have baseline knowledge of being able to perform CPR or how to operate an AED if asked. After vitals are obtained, patient Mizuno will state, “Something is wrong with my leg; I can’t move it!” He will also state numerous times, “What happened--why is everybody hurt?” but will be able to be reassured by participants. Patient Ping will also be upset, stating, “My arm keeps bleeding! I feel so dizzy! What happened?” He will also be consolable if given sufficient attention. Neither of these two patients will initially notice their unconscious colleague. The purpose of these statements is to provide a mild distraction from the provider’s primary objective, which is resuscitating patient Callaway. If the AED is applied to patient Callaway, it will demonstrate PEA. The providers should administer appropriate interventions, such as performing a jaw thrust, CPR, and providing rescue breaths. If the patient is given an erroneous defibrillation for PEA, return of spontaneous circulation (ROSC) will not be achieved. If the patient does not receive rescue breaths, ROSC will not be achieved. After a total of 6 minutes, if patient Mizuno is ignored, he will become upset with a raised voice to serve as an increasing distraction. He will be able to be calmed if given sufficient reorientation and reassurance. Explaining to him that his symptoms are most likely temporary, and he will recover without treatment will reassure him and encourage him to remain calm. If patient Mizuno becomes upset, patient Ping will mirror his emotional state approximately one minute later and be unable to be consoled, providing further confusion and distraction in the scenario. If patient Mizuno is treated promptly with evaluation and reassurance, he will become more cooperative as he becomes more aware of the global situation. Likewise, patient Ping should also become more reasonable especially once ROSC for patient Callaway is achieved. This scenario will be considered successful if the team is able to resuscitate patient Callaway with rescue ventilation and CPR, to identify and educate patient Mizuno regarding his keraunoparalysis, to apply a tourniquet to patient Ping’s upper extremity, and if emergency medical services are contacted. An optimal outcome would include a MARCH evaluation for each patient (Massive hemorrhage, Airway with C-Spine considerations, Respiratory management, Circulation, Hyper/Hypothermia/Hike/Helicopter)^11^ with dispositions for each. The length for all of these measures to be achieved should be approximately 15 minutes for a 3- to 4-person team.

Non-ideal management by the residents should include failure to treat patient Callaway first, defibrillating patient Callaway, not providing respiratory support for patient Callaway, failure to correctly evaluate and reassure patient Mizuno, improper tourniquet placement, or not providing correct disposition by failing to contact EMS.

**Critical actions:**

1. Assess circulation, airway, and breathing on all three patients
2. Treatment via reverse triage, prioritizing resuscitation of the unresponsive, pulseless patient
3. Recognition of keraunoparalysis and educating the patient that this will self-resolve
4. Application of a tourniquet to the penetrating arterial injury
5. Calling Emergency Medical Services for disposition

**Case Title:** Lightning Strike Patient Taylor Callaway

**Chief Complaint:** Patient is Taylor Callaway, a 35-year-old male, who presents at the scene post lightning strike.

**General Appearance:** Unresponsive

**Primary Survey:**

- **Airway:** intact
- **Breathing:** No spontaneous respirations
- **Circulation:** no pulses

**History:**

- **History of present illness:** Patient is Taylor Callaway, who is a 35-year-old male who presents as an unresponsive patient post lightning strike. He was playing a game of golf with two friends when a lightning strike from an approaching thunderstorm struck the group and rendered him unresponsive.
- **Past medical history:** Unknown
- **Past surgical history:** Unknown
- **Patient’s medications:** Unknown
- **Allergies:** Unknown
- **Social history:** Unknown
- **Family history:** Unknown
- **Vital signs**:
  ○ HR – 0
  ○ Resp rate – No spontaneous respirations.
  ○ Temp – Normothermic to touch
- **Weight:** 220 lbs., 100 kg
- **Assessment:** Patient is unresponsive, pulseless, and does not have any respiratory effort.

**Secondary Survey/Physical Examination:**

- **General appearance:** Unresponsive
- **HEENT:**
  ○ **Head:** within normal limits (wnl)
  ○ **Eyes:** fixed, dilated pupils
  ○ **Ears:** wnl
  ○ **Nose:** wnl
  ○ **Throat:** wnl
- **Neck:** wnl (within normal limits)
- **Heart:** No heart sounds
- **Lungs:** No spontaneous respiratory effort
- **Abdominal/GI:** wnl
- **Genitourinary:** deferred
- **Rectal:** deferred
- **Extremities:** Lichtenberg figure from the right proximal arm down to the wrist
- **Back:** wnl
- **Neuro:** reflexes intact
- **Skin:** Lichtenberg figure from the right proximal arm down to the wrist
- **Lymph:** wnl
- **Psych:** Unable to assess

**Results:**

*Lichtenberg Figure on Skin*

Heilman J. A person who was affected by a nearby lightning strike. Note the slight branching redness traveling up his leg from the effects of the current. In: Wikimedia Commons. https://commons.wikimedia.org/wiki/File:Lightning_injury.jpg Published Aug 14, 2019. CC BY-SA-3.0

## OPERATOR MATERIALS

**Diagnosis:**

PEA arrest status post lightning strike

**Disposition:**

Call EMS for ED evaluation

**Case Title:** Lightning Strike Patient Nike Mizuno

**Chief Complaint:** Patient is Nike Mizuno, a 37-year-old male, who presents at the scene post lightning strike.

**General Appearance:** conversational but distraught, sitting on the ground

**Primary Survey:**

- **Airway:** intact
- **Breathing:** Tachypneic
- **Circulation:** HR 120, right lower extremity is blue and mottled, cold to the touch (verbalized if checked), paralytic and pulseless

**History:**

- **History of present illness:** Patient is Nike Mizuno, who is a 37-year-old male who presents with keraunoparalysis post lightning strike. He was playing a game of golf with two friends when a lightning strike from an approaching thunderstorm struck the group giving him right lower extremity cyanosis and paralysis.
- **Past medical history:** Depression
- **Past surgical history:** none
- **Patient’s medications:** Sertraline 10mg
- **Allergies:** none
- **Social history:** Non-contributory
- **Family history:** Non-contributory
- **Vital signs**:
  ○ HR – 120
  ○ Resp rate – 22 breaths per minute
  ○ Temp – Normothermic to touch other than right lower leg
- **Weight:** 200 lbs., 91 kg
- **Assessment:** Patient is unresponsive, pulseless, and does not have any respiratory effort.

**Secondary Survey/Physical Examination:**

- **General appearance: Upset**, difficult to redirect from injury.
- **HEENT:**
  ○ **Head:** within normal limits (wnl)
  ○ **Eyes:** wnl, normal pupils
  ○ **Ears:** wnl
  ○ **Nose:** wnl
  ○ **Throat:** wnl
- **Neck:** wnl
- **Heart:** Regular tachycardic rhythm
- **Lungs:** wnl, mildly tachypneic at rest
- **Abdominal/GI:** wnl
- **Genitourinary:** deferred
- **Rectal:** deferred
- **Extremities:** Cyanosis with mottling and 1/5 strength with decreased sensation in the right lower extremity. Sensation is decreased in all dermatomes. No dorsalis pedis or posterior tibial pulse palpable. All lower extremity compartments are soft and compressible. All other extremities wnl
- **Back:** wnl
- **Neuro:** as above for right lower extremity.
- **Skin:** right lower extremity is cool to the touch and cyanotic with mottling
- **Lymph:** wnl
- **Psych:** Concerned about weakness and paresthesia in right leg

**Diagnosis:**

Lightning Strike Patient, keraunoparalysis

**Disposition:**

EMS transport to the ED for overnight observation

**Case Title:** Lightning Strike Patient Wilson Ping

**Chief Complaint:** Patient is Wilson Ping, who is a 30-year-old male who presents with a penetrating arterial arm injury post lightning strike.

**General Appearance:** In pain, difficult to redirect from injury

**Primary Survey:**

- **Airway:** intact
- **Breathing:** Tachypneic
- **Circulation:** HR 135, thready left radial pulse

**History:**

- **History of present illness:** Patient is Wilson Ping, who is a 30-year-old male who presents with a penetrating arterial arm injury post lightning strike. He was playing a game of golf with two friends when a lightning strike struck the group, causing a piece of wood to lodge into his left upper arm
- **Past medical history**: Unremarkable
- **Past surgical history:** none
- **Patient’s medications:** Daily multivitamin
- **Allergies:** none
- **Social history:** Non-contributory
- **Family history:** Non-contributory
- **Vital signs**:
  ○ HR – 135
  ○ Resp rate – 20 breaths per minute
  ○ Temp – normothermic to touch
- **Weight:** 200 lbs., 91 kg

**Assessment:** Patient has active arterial bleeding from a penetrating injury to his left upper arm with a thready distal pulse

**Secondary Survey/Physical Examination:**

- **General appearance:** In pain, difficult to redirect from injury
- **HEENT:**
  ○ **Head:** within normal limits (wnl)
  ○ **Eyes:** wnl, normal pupils
  ○ **Ears:** wnl
  ○ **Nose:** wnl
  ○ **Throat:** wnl
- **Neck:** wnl
- **Heart:** Regular tachycardic rhythm, thready left radial pulse
- **Lungs:** Mildly tachypneic at rest
- **Abdominal/GI:** wnl
- **Genitourinary:** deferred
- **Rectal:** deferred
- **Extremities:** active arterial bleeding from penetrating wound to the left arm. Thready left radial pulse. No bony deformity.
- **Back:** wnl
- **Neuro:** alert and oriented x3
- **Skin:** as above for extremities, otherwise wnl
- **Lymph:** wnl
- **Psych:** anxious

**Diagnosis:**

Lightning Strike, penetrating arterial injury

**Disposition:**

Transport to the emergency department for further definitive management

## DEBRIEFING AND EVALUATION PEARLS

### Lightning Strike

#### Learning Points

1. Establishing scene safety for providers is the critical initial step
2. Most lightning strike patients die from respiratory arrest from paralysis of the medulla; beginning rescue breaths immediately and starting quality CPR is of critical importance
3. Triage lightning strike patients in a “reverse triage” fashion with pulseless, apneic patients treated first. This may help save lives in those in respiratory arrest who are likely to have good outcomes with expedient ventilatory support
4. Lighting strikes can result in unusual injuries such as blast injuries and keraunoparalysis
5. Assessing availability and appropriate utilization of limited resources is essential to ensuring patients receive the appropriate amount of care they need in a timely manner (crisis resource management)

#### Pearls

- The most important consideration in the treatment of lightning casualties is to ensure the safety of the providers. Getting to the shelter of a large building with interior rooms or a closed metal vehicle will offer the best safety.^5^
- If unable to move indoors, avoid tall objects or exposed ground; lightning will tend to strike taller objects. Squat crouched to the ground with your feet together and contacting the ground to reduce your exposure to ground current.^5^,^6^
- The highest risk of lightning strike injury is 20 minutes before and after a storm.
- If traveling in a group, spread out to at least 7 meters from one another but within eyesight if caught in the open from a lightning storm.
- For lightning, reverse triage is performed where pulseless and apneic patients are treated before other patients; this is the opposite of other triage protocols and is commonly referred to as the “reverse triage” technique.^5^,^6^ The reason for performing reverse triage is that in traditional triage systems, patients who appear to be dead (pulseless and apneic) are tagged black (dead) and de-prioritized. However, in a lightning strike mass casualty, patients who are pulseless and apneic without other fatal injuries can often be resuscitated with BLS (Basic Life Support) including ventilation.
- Lightning cannot be compared to other types of electrical injury because it is a large pulse of ionized energy, producing a unique injury pattern when compared to standard electrocutions.^6^
- Lightning strikes victims in one of six ways; direct strike, side splash, contact, ground current, upward streamers, or blunt trauma.^1^
  ○ Direct strike is when a person is impacted by the bolt directly, causing the most devastating injuries. This is the most fatal type of lightning strike.
  ○ Side-splash is when a person receives the current from a nearby object which has sustained the direct strike; it is possible for side splash from one person to incapacitate several people.^7^
  ○ Contact exposures occur when a patient is in physical contact with an object sustaining a direct strike, such as a fishing rod, golf club, or umbrella.
  ○ Ground current strikes occur when the lightning bolt electrifies the wet ground around the victim(s), causing electrical current to travel up the leg. This is another common cause of mass strike events.^7^
  ○ Upward streamer strikes are caused by skyward climbing columns of charge trying to complete the circuit between the sky and the ground and can electrify humans if they become part of the circuit. These injuries are typically less severe.
  ○ Blunt trauma is always a consideration because the energies involved in lightning strikes can be tremendous, with sufficient energy to flash-heat water into steam, causing localized explosions when wet clothing or trees are disintegrated by this process^3^. The force of this can also cause blunt trauma such as fractures, traumatic brain injuries, or over-pressure type injuries.
  ○ Blast injuries are typically categorized into primary, secondary, tertiary, and quaternary injuries.^8^
    ▪ Primary blast injuries affect gas-filled structures, causing such injuries such as tympanic membrane perforation, globe rupture, gastrointestinal perforation, blast lung, and pneumothorax
    ▪ Secondary blast injuries are caused by airborne shrapnel as they come into contact with a patient.
    ▪ Tertiary blast injuries occur from a patient’s body being thrown due to blast wind or collapse of surrounding structures. Resultant blunt or crush injuries may occur.
    ▪ Quaternary blast injuries describe burns and radiation exposure.
  ○ Wilderness training utilizes the MARCH treatment algorithm; it stands for Massive hemorrhage, Airway/C-spine, Respirations, Circulation, Hypo/Hyperthermia, Hike/Helicopter.^5^ In the setting of lightning injuries, rescue respirations are the most important first intervention to overcome the medullary paralysis. Compressions may be required, as is the case here, if the heart does not immediately restart.
- Lightning injures effect many organ systems, including the central nervous system (primary cause of death), the peripheral and autonomic nervous system, the cardiovascular system, the integumental system, the musculoskeletal system, the ocular system, and the auditory system
  ○ In most cases, the cause of death in a lightning strike is from paralysis of the medulla resulting in respiratory arrest.^1^ This is why CPR with rescue breathing should be initiated immediately. Early CPR is the motivator for the reverse triage protocol.^1^
  ○ The cardiovascular system is likewise thrown into disarray with various atrial and ventricular dysrhythmias documented.^2^ Initially, victims generally experience asystole by the surge of electricity, but the heart will eventually start beating on its own from inherent automaticity.^1^ A prolongation of the QTc is not uncommon and dysrhythmias can persist for months.^7^
  ○ Keraunoparalysis is an unusual lightning-specific injury which presents as single or multiple limbs becoming pulseless and cyanotic with subsequent paralysis. This condition is temporary, resolving in as little as 30 minutes usually without long-term sequela. 9 Patients describe a numb or paresthetic limb with greatly reduced strength. The pathophysiology of this condition is thought to be caused by an catecholamine surge from the effected autonomic nerves in the limb, causing arterial vasoconstriction and the signs and symptoms noted above.^9^
  ○ The skin often demonstrates some stigmata of lightning injury in direct strikes, with painless Lichtenberg figures being the most common. These are not true burns, but rather alterations of skin pigmentation which diminish soon after the strike.^10^ Superficial and thermal burns from the strike itself, steam produced by wet clothing, and heated metal clothing items such as necklaces, wristwatches, and zippers are all burns that the victim should be examined for. These can be treated under a traditional thermal burn algorithm.
  ○ Retrograde amnesia is commonly observed in lightning strike victims. These patients may need frequent re-direction and may be disoriented due to not being able to remember the immediate events surrounding the lightning strike.
  ○ The musculoskeletal system may experience myonecrosis and fractures from either the muscular contortion of the electrical discharge of the strike or from the blunt trauma. Myonecrosis is unusual in lightning injuries when compared to high voltage electrical exposures, but has been documented.^10^
  ○ The eyes are commonly injured, even in minor lightning strikes, with cataracts often forming in affected persons. Dilated and unreactive pupils are a possibility in the initial paralysis phase after a strike, but this should not dissuade one from performing CPR or be used to determine brain death.^1^
  ○ Deafness from the report of the strike or tympanic membrane disruption from the pressure blast are common lightning injuries. As many as 50% of lightning victims suffer from tympanic membrane rupture in at least one ear.^11^
- Long term effects of lightning strikes are well documented and are primarily cardiovascular, neurological, and behavioral in nature. Sleep disturbance, personality changes, depression, and cardiac dysrhythmias are all common sequelae.^10^,^12^

**Other debriefing points:**

Closed-loop communication amongst team: Was it used? Why or why not? Were there any implications of this during case execution?

## SIMULATION ASSESSMENT

*Lightning Strike*

Learner: _________________________________________

***Assessment Timeline***

This timeline is to help observers assess their learners. It allows observer to make notes on when learners performed various tasks, which can help guide debriefing discussion.

**Critical Actions:**

□ Assess circulation, airway, and breathing on all three patients
□ Treatment via reverse triage, prioritizing resuscitation of the unresponsive, pulseless patient
□ Recognition of keraunoparalysis and educating the patient that this will self-resolve
□ Application of a tourniquet to the penetrating arterial injury
□ Calling Emergency Medical Services for disposition

**Summative and formative comments:**

**Milestones assessment:**
