# Supplementary figures and images for: Lightning Strike
Source: J Educ Teach Emerg Med. 2022 Jan 15;7(2):S78–S106. doi: 10.21980/J8SD2M (PMC10332745; doi:10.21980/J8SD2M)

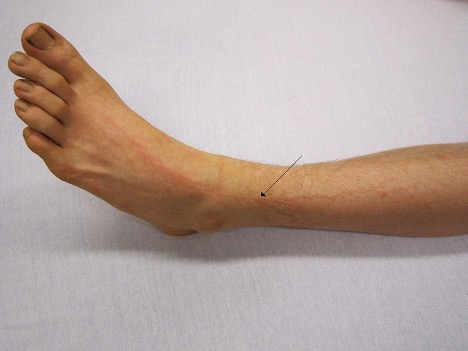

Supplement: Supplementary file 1 [file JETem-7-2-S78-supp1.jpg]
